# Detecting dementia in patients with normal neuropsychological screening by Short Smell Test and Palmo-Mental Reflex Test: an observational study

**DOI:** 10.1186/s12877-015-0094-0

**Published:** 2015-07-25

**Authors:** Sven Streit, Andreas Limacher, Andreas Zeller, Markus Bürge

**Affiliations:** Institute of Primary Health Care BIHAM, University of Bern and Geriatrics University of Bern, Inselspital and Spital Netz Bern, Bern, Switzerland; Clinical Trials Unit Bern, Department of Clinical Research, and Institute of Social and Preventive Medicine, University of Bern, Bern, Switzerland; Centre for Primary Health Care, University of Basel, Basel, Switzerland; Geriatrics University of Bern, Inselspital and Spital Netz Bern AG Spital Ziegler Morillonstrasse, 75-91 3001 Bern, Switzerland

**Keywords:** Dementia, Additional testing for dementia in cases of negative neuropsychological screening, Short Smell Test (SST), Palmo-Mental Reflex (PMR)

## Abstract

**Background:**

General practitioners (GPs) are in best position to suspect dementia. Mini-Mental State Examination (MMSE) and Clock Drawing Test (CDT) are widely used. Additional neurological tests may increase the accuracy of diagnosis. We aimed to evaluate diagnostic ability to detect dementia with a Short Smell Test (SST) and Palmo-Mental Reflex (PMR) in patients whose MMSE and CDT are normal, but who show signs of cognitive dysfunction.

**Methods:**

This was a 3.5-year cross-sectional observational study in the Memory Clinic of the University Department of Geriatrics in Bern, Switzerland. Participating patients with normal MMSE (>26 points) and CDT (>5 points) were referred by GPs because they suspected dementia. All were examined according to a standardized protocol. Diagnosis of dementia was based on DSM-IV TR criteria. We used SST and PMR to determine if they accurately detected dementia.

**Results:**

In our cohort, 154 patients suspected of dementia had normal MMSE and CDT test results. Of these, 17 (11 %) were demented. If SST or PMR were abnormal, sensitivity was 71 % (95 % CI 44–90 %), and specificity 64 % (95 % CI 55–72 %) for detecting dementia. If both tests were abnormal, sensitivity was 24 % (95 % CI 7–50 %), but specificity increased to 93 % (95 % CI 88–97 %).

**Conclusion:**

Patients suspected of dementia, but with normal MMSE and CDT results, may benefit if SST and PMR are added as diagnostic tools. If both SST and PMR are abnormal, this is a red flag to investigate these patients further, even though their negative neuropsychological screening results.

**Electronic supplementary material:**

The online version of this article (doi:10.1186/s12877-015-0094-0) contains supplementary material, which is available to authorized users.

## Background

General practitioners (GPs) are in a good position to detect dementia early. Cognitive dysfunction may be reported by patients, caregivers, or relatives, or discovered during general screening (e.g. while testing fitness to drive). Early detection of dementia can reduce the burden on the caregiver through effective intervention [1], and can delay admission to a nursing home [2], and mitigate the behavioural and psychological symptoms of dementia. At this early stage, patients can also state preferences for care, which may lower the rate of unwanted hospitalisation [3].

GPs often use the Mini-Mental State Examination (MMSE) and the Clock Drawing Test (CDT) to test for dementia. The CDT is popular because it assesses multiple domains, particularly executive functions, based on a single task. In Switzerland, the National Association of Swiss Memory Clinics (SMC) [http://www.swissmemoryclinics.ch last access July 15th, 2015] endorses using MMSE and CDT [4]. Screening indications are self-reported cognitive dysfunction or suspicion for dementia by caregivers or relatives, but it is challenging to detect dementia in its early stages. Studies show that the rate at which these tests fail to identify dementia patients is variable (MMSE 3 %–26 %, depending on the cut-off [5, 6]; CDT alone 25 %) [7]. Combining MMSE (cut off ≤24/30 points, which is very low) and CDT returned a false negative rate of 9 % [8]. If GPs can use easy and practical neurological tests to detect dementia, they may be able to more accurately judge whether a patient needs further investigation.

GPs refer patients suspected of dementia to Memory Clinics, which were established to further examine patients whose screening test results were negative, but who had abnormal cognitive test results or unexplained cognitive dysfunction [4]. Memory Clinic staff conduct in-depth interviews with patients and relatives or caregivers and use additional neuropsychological tests to diagnose dementia. Neither the Short Smell Test (SST) nor the Palmo-Mental Reflex (PMR) test have been used to detect dementia, but we chose SST and PMR because they turn deficient early in various diffuse subcortical or cortical encephalopathies that often lead to dementia: Stamps et al. found that patients who could not detect the odour of peanut butter with their left nostril were likely to have Alzheimer’s disease [9]. In a review, Barresi et al. found evidence that olfactory dysfunction is involved in various neurological diseases [10]. PMR has been described in detail in patients with dementia [11]. This primitive reflex is often present in diffuse diseases like subcortical encephalopathies (with white matter changes) [12] or more cortical encephalopathies (such as Alzheimer’s Disease) [13].

Our study focused on patients whose results on the MMSE and CDT were negative, but who were admitted to a Swiss Memory Clinic because of suspected dementia. We used a cross-sectional observational study to determine if adding SST and PMR tests increased the chances of dementia would be detected in patients whose MMSE and CDT were normal.

## Methods

### Population

Our study was conducted in the Memory Clinic of the University Department of Geriatrics in Bern, Switzerland, one of over 37 Memory Clinics in Switzerland. According to national quality standards, dementia is diagnosed in every Swiss Memory Clinic by an expert team, based on national and international guidelines. Every clinic sees at least 100 outpatients each year. Because each Memory Clinic records medical data differently, we located our study in a single Memory Clinic that sees about 200 patients a year. Of these, about 60 % are diagnosed with dementia.

Patients were referred by GPs who sought diagnostic clarification, and who looked to the Memory Clinic to detect and characterize underlying cognitive disorders. Patients may have self-reported their concerns about their mental state, relatives may have reported concerns, or patients may have had abnormal results on screening tests for dementia. We included patients given MMSE and CDT tests in the Memory Clinic, whose results on these tests were normal. Test results were normal if MMSE was ≥27 out of 30 points (as suggested by Monsch et al. [6]) and CDT ≥6 out of 7 points (as suggested by Thalmann et al. [14]; for detailed description see Table 2 in that article). We did not exclude any patients. We retrospectively collected data from the medical records of all consecutive patients admitted to this Memory Clinic between May 2009 and December 2012. The Ethics Committee of Bern approved the study. The National Confederate Expert Panel for Professional Secrecy permitted us to use personalized data.

### General assessment

Residents in General Internal Medicine and Geriatrics interviewed patients and clinically evaluated them under the supervision of an attending physician. A neuropsychologist supervised the neuropsychological tests. Geriatricians in Switzerland are specialists in general internal medicine, and have also completed a 3-year curriculum in geriatrics and psychiatry. Neuropsychologists have a Masters in psychology (MSc in Psychology).

Patients were assessed in two sessions, based on a standardized protocol. In the first session, semi-structured interviews were conducted with patients and, if possible, at least one close relative or caregiver. Interviews covered the following topics: personal data, education, reason for admission, cognitive complaints, behavioural and psychological symptoms of dementia (BPSD), problems in practical daily routines, somatic problems, earlier medical problems, family medical and social history. The interviewer assessed basic and instrumental activities of daily living (BADL, IADL) and applied the Nurses’ Observation Scale for Geriatric Patients (NOSGER II). The clinical examination checked for neurological abnormalities, and also included the Short Smell Test (SST) and Palmo-Mental Reflex (PMR), both of which are described in detail in the next subsection. If the admitting GP had not already ordered cerebral images and laboratory tests, the Memory Clinic did these at the end of the first session. In the second session, patients were given a standardised battery of neuropsychological tests, including the Consortium to Establish a Registry for Alzheimer’s Disease (CERAD Plus) [15] and supplementary tests. (Additional file 1).

### Detailed description of short smell test (SST) and Palmo-Mental Reflex (PMR)

Simple SST was considered abnormal if patients closed their eyes and could not identify instant coffee powder in a can when it was held 5–10 cm under their nose.

PMR was considered positive if brushing the thumb under the thenar (the region of the palm at the base of the thumb) elicited a unilateral chin muscle twitch. (See the Youtube video we recorded to demonstrate the test [16]).

### Standard diagnosis of dementia and its aetiology

After all the data is collected, dementia is diagnosed at the Memory Clinic in two-steps. First, a team of experts (senior consultant, neuropsychologist) diagnoses dementia according to gold standard DSM-IV TR criteria [17]. The team uses criteria set by the International Working Group on Mild Cognitive Impairment to diagnose mild cognitive impairment (MCI, defined as patients in cognitive decline, who can still conduct basic and instrumental activities of daily living, and do not meet DSM-IV TR criteria for dementia) [18]. SST and PMR are not considered in this first step, because dementia is not diagnosed based on deficiencies revealed by SST and PMR. In the second step, the team of experts assigns probable aetiology (e.g. for Alzheimer’s disease, fronto-temporal dementia, dementia with Lewy bodies, vascular dementia, mixed or other types of dementia) based on published common consensus criteria for the specific subtype of dementia. At this point, experts may use SST and PMR results only to assist in determining the aetiology of dementia.

### Assessing diagnostic accuracy of SST and PMR: retrospective statistical analysis

Means and standard deviations or numbers and proportions were used for descriptive statistics. We compared categorical data with the chi-squared test and continuous data with the *t*-test. To test inter-rater reliability, two residents of the Memory Clinic independently administered the PMR to a consecutive sample of patients (*n* = 42) after the retrospective study.

To test diagnostic accuracy of SST and PMR, we calculated the sensitivity, specificity and likelihood ratios (LR), Receiver Operating Characteristic (ROC), and the area under the ROC curve (AUC). In the first model, either SST or PMR had to be abnormal to detect dementia. In the second model, both SST and PMR had to be abnormal to detect dementia. To adjust for the fact that patients with dementia were significantly older, we stratified them into two groups: younger and older patients.

We used STATA release 13.1 (Stata Corp, College Station, TX, USA), and considered a *p*-value of 0.05 to be statistically significant.

## Results

### Baseline characteristics

**Fig. 1:**
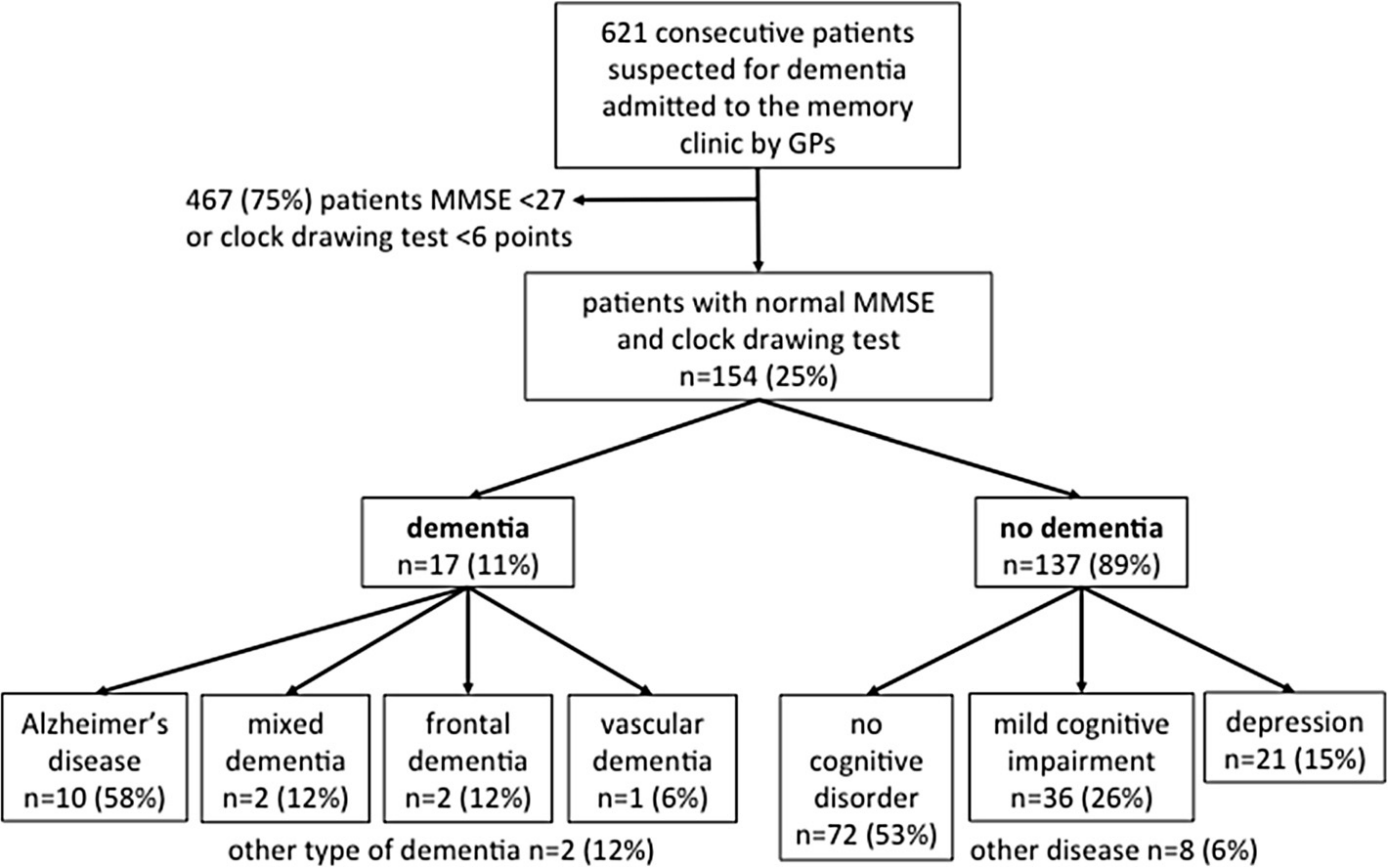
Patient flow chart

During the observation period of 3.5 years, our Memory Clinic admitted 621 patients (Fig. 1). Of these, 62 % were diagnosed with dementia. Residents worked at the Memory Clinic on a 3-month rotation, so the staff changed over time. The senior physician and neuropsychologist were the same throughout.

We included 154 patients in our study whose basic examination fell within the normal range (MMSE ≥27 points *and* CDT ≥6 points). We detected 17 (11 %) cases of dementia (Table 1). Patients with dementia were significantly older than patients without dementia (p > 0.001); there was no gender difference (*p* = 0.3; data not shown). Types of dementia included Alzheimer’s disease (58 %), mixed dementia (12 %), frontal dementia (12 %), vascular dementia (6 %), or other type of dementia (12 %). No dementia was found in 137 patients (89 %). There were no objective findings for most of these patients with complaints (53 %). Some had MCI (26 %), depression (15 %), or another disease (6 %) (Table 1). SST was abnormal in 53 % of all patients with dementia, and abnormal in 25 % of all patients without dementia (*p* = 0.015). PMR was abnormal in 41 % of all patients with dementia, and abnormal in 18 % of all patients without dementia (*p* = 0.028). There were also significant differences between patients with and without dementia for MMSE and CDT, SST, PMR); *p*-values were between 0.04–0.004.

**Table 1:** Baseline characteristics

Baseline characteristics	Overall (*n* = 154)
Patient characteristics	
Age, mean (SD)	68.5 (11)
Women, n (%)	81 (53)
Dementia, n (%)	17 (11)
Alzheimer’s disease	10 (58)
Mixed dementia	2 (12)
Frontal dementia	2 (12)
Vascular dementia	1 (6)
Other type of dementia	2 (12)
No dementia, no (%)	137 (89)
Complaints but no objective findings	72 (53)
Mild cognitive impairment (MCI)	36 (26)
Depression	21 (15)
Other disease	8 (6)
Test results	
MMSE^a^, mean (SD)	28.5 (1.1)
CDT^b^, mean (SD)	7.0 (0.2)
Abnormal SST^c^, n (%)	43 (28)
Positive PMR^d^, n (%)	32 (21)
Abnormal SST and positive PMR, n (%)	13 (8)

^a^Mini-Mental state examination

^b^Clock drawing test

^c^Short Smell Test

^d^Palmo-Mental Reflex

Inter-rater reliability of PMR testing substantially agreed (kappa 0.80, 95 % -CI 0.62 - 0.99, data not shown).

### Diagnostic performance of SST and PMR

**Fig. 2:**
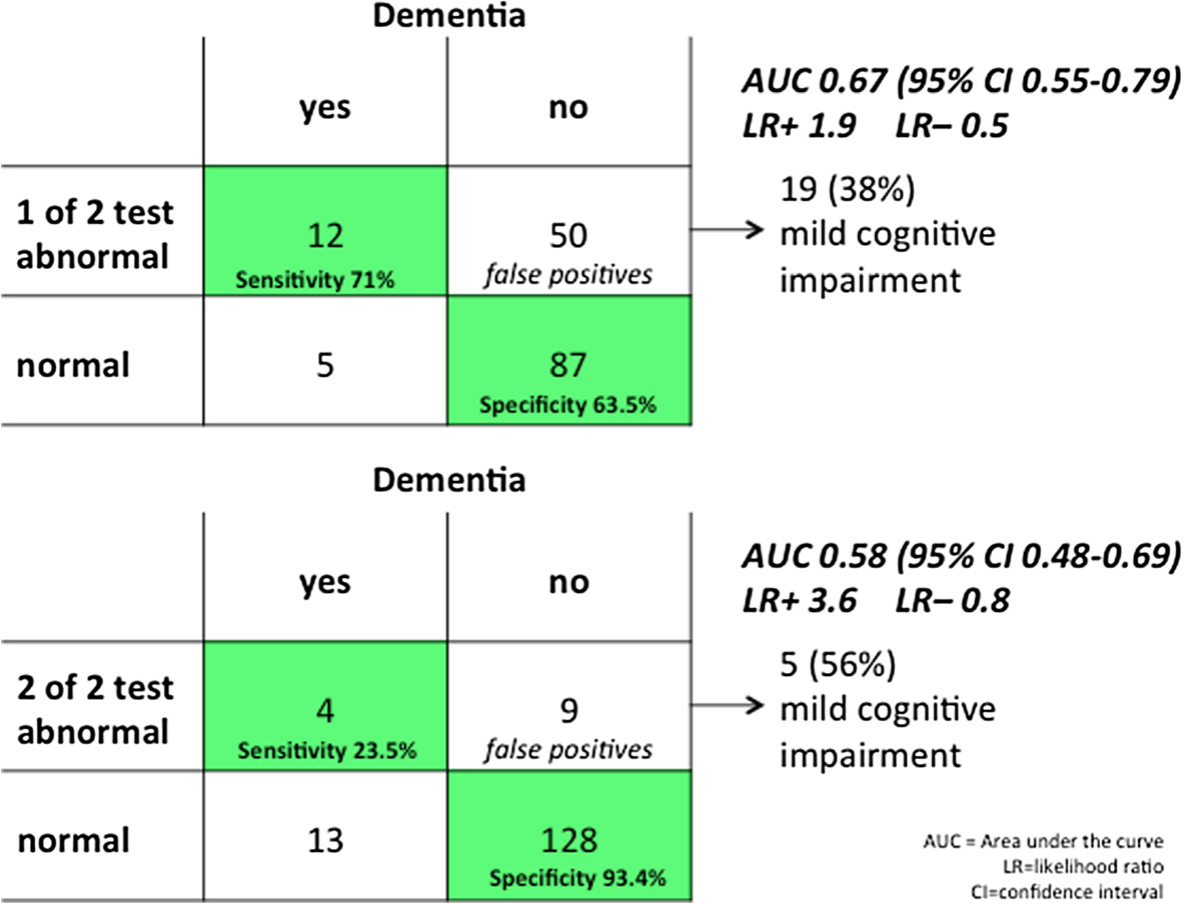
Diagnostic performance in detecting dementia by abnormal smell and Palmo-Mental Reflex in patients with normal MMSE (≥27) or CDT (≥6 points). One of 2 tests abnormal (upper chart) or both abnormal (lower part) contributed to the diagnosis of dementia

The first model shows that patients with at least one abnormal SST or PMR test were more likely to be demented (Fig. 2): Sensitivity was 71 % (95 % CI 44-90 %), and specificity 64 % (95 % CI 55-72 %) for detecting dementia. Among the 50 false positives, 19 patients (38 %) had an MCI.

The second model shows that patients whose SST and PMR are both abnormal are much more likely to have dementia (Fig. 2). Specificity was very high (93 % [95 % CI 88-97 %]), but sensitivity was low (24 % [95 % CI 7-50 %]). Of the 9 false positives, 5 patients (56 %) had MCI.

Since patients with dementia were significantly older (Table 1), we stratified the second model into groups aged >70 and <70 years (data not shown). Specificity in patients aged <70 years was 96 % (95 % CI 88-99 %) and 90 % (95 % CI 80-96 %) in patients >70 years of age.

## Discussion

SST and PMR were both helpful in detecting dementia in patients with normal MMSE and CDT results. Dementia was present in over 93 % of patients whose SST and PMR were both abnormal. A high percentage of patients with false positive results on the SST and PMR (38 %-56 %) had MCI results that justified referral to a specialized Memory Clinic.

As far as we can determine, this was the first study to combine these two neurological tests with neuropsychological screening by the GP. Studies such as by Kato Y et al. [8] described the combination of MMSE and CDT as a powerful screening tool with high sensitivity and specificity (89.4 vs. 83.9 %). Kato’s study is one of the few studies to examine the tests in combination in a sample of hospitalized patients. Devanand DP et al. found that poor scores on smell tests predicted cognitive decline in 1,037 patients in a community cohort in New York, USA. This study suggests SST is an early biomarker of cognitive decline in patients with Alzheimer’s disease [19]. Stanciu I et al. had similar results after following a Swedish cohort of 1,529 patients. Low scores on odour identification tests independently predicted conversion to dementia [20].

### Limitations

Our sample size was small (154 patients), so the confidence interval is wide, although we collected our samples over a long observation period of 3.5 years. Although it is plausible that loss of smell is related to dementia [21], abnormal SST can also be caused by other physiological changes like decreased hydration and thinning of the olfactory mucosa [22]. Normal aging may account for the increase in abnormal SST and PMR results, though our sensitivity analysis showed that younger and older patients had similar high specificity, regardless of gender. Infections or other pathologies may have impaired sense of smell, even though patients and relatives were advised to shift their appointments if they had acute infections or their general condition significantly deteriorated.

Some dismiss PMR because the methods used to elicit results, and their interpretation are inconsistent [23]. We addressed this by standardizing PMR testing in our study. We asked an experienced physician to teach new colleagues how to administer the test. Thus, the prospective inter-rater reliability test showed substantial agreement. Without good instructions PMR testing would be difficult to successfully implement PMR testing in primary care. We provide a teaching video that demonstrates the way we performed the PMR test [16]. Though patients receive both SST and PMR tests as part of the standardized protocol, and results are available to the expert team when they diagnose dementia, the gold standard for diagnosing dementia includes neither the SST nor the PMR.

### Strengths

The standardized tests at our Memory Clinic gave us very high quality data: none were missing. Our sample of patients can be generalized to a broader population because of its similar distribution of types of dementia and other diagnoses beside dementia, such as depression. The relatively high cut-off we set for MMSE testing (27 or more points judged as normal) reduced false negative results, though using a lower cut-off would have made SST and PMR even more sensitive.

Our findings indicate that both SST and PMR should be validated in a primary care setting that ideally offers other screening tests than MMSE and CDT as well. We recommend that patients suspected of dementia, who have abnormal SST and PMR, be evaluated further, even if they have normal MMSE and CDT results. GPs should see abnormal SST and PMR as a red flag that may indicate dementia. If one or both tests are normal, we do not suggest that a change in current practice is necessary: patients, caregivers and doctors should together decide the next steps to take. However, abnormal results on both tests indicate that further testing is warranted.

## Conclusions

For patients whose neuropsychological screening (MMSE, CDT) is normal, but who are suspected of dementia, PMR and SST offer additional diagnostic value. Because specificity is very high (93 %), we suggest abnormal results on both the PMR and SST are a red flag for GPs, indicating patients should be further evaluated. Mild cognitive impairment was common among individuals with false positive results for PMR and SST, so we recommend further testing. Adding PMR and SST to dementia evaluations may help physicians to detect dementia early, and reduce its burden through early intervention.

## Additional file

Additional file 1:
**Standard procedure for the evaluation of dementia at the Memory Clinic of the Department of Geriatrics, (Inselspital, Bern University Hospital, and) Spital Netz Bern Ziegler, and University of Bern, Switzerland.** (PDF 46 kb)
