# Supplementary material for: Detecting dementia in patients with normal neuropsychological screening by Short Smell Test and Palmo-Mental Reflex Test: an observational study
Source: BMC Geriatr. 2015 Jul 25;15:90. doi: 10.1186/s12877-015-0094-0 (PMC4513974; doi:10.1186/s12877-015-0094-0)
Supplement: Additional file 1: — Standard procedure for the evaluation of dementia at the Memory Clinic of the Department of Geriatrics, (Inselspital, Bern University Hospital, and) Spital Netz Bern Ziegler, and University of Bern, Switzerland. (PDF 46 kb) [file 12877_2015_94_MOESM1_ESM.pdf]

Appendix Table 1

Standard procedure for the evaluation of dementia at the Memory Clinic of the Department of Geriatrics, (Inselspital, Bern University Hospital, and) Spital Netz Bern Ziegler, and University of Bern, Switzerland

Clinical interview with patient and caregiver

Standardized questions regarding the following topics are given to both the patient and the caregiver:

Reason for referral
Demographic information
Medical and social history
Cognitive symptoms (e.g. memory, orientation, attention, language, problem solving, planning, performing routine tasks)
Emotional symptoms (e.g. depressed mood, apathy, irritability, hallucinations, inappropriate behavior)
Physical symptoms (e.g. gait, balance, falls, smell, dizziness, tremor, sleep)
Basic activities of daily living BADL
Instrumental activities of daily living IADL

Questionnaire to be filled out by the caregiver:

Nurses' Oberservation Scale for Geriatric Patients NOSGER II

Clinical examination

Weight, height, vital signs
Neurological examination based on a standardized protocol
Geriatric evaluation of gait and balance (Get Up and Go Test)
Additional medical examination if necessary

Further diagnostic examinations if not already performed

Imaging of the brain (MRI or CT)
Blood tests

Neuropsychological assessment

Core dementia assessment battery: CERAD plus (Consortium to Establish a Registry for Alzheimer's Disease) completed by neuropsychological tests in the following domains: auditory and visual attention, working memory, abstract thinking, judgment, oral comprehension, mental and written calculation, visual-perceptual skills, apraxia.
Depression screening: GDS 15 items (Geriatric Depression Scale)
